# Health literacy–listening skill and patient questions following cancer prevention and screening discussions

**DOI:** 10.1111/hex.12387

**Published:** 2015-07-22

**Authors:** Kathleen M. Mazor, Donald L. Rubin, Douglas W. Roblin, Andrew E. Williams, Paul K. J. Han, Bridget Gaglio, Sarah L. Cutrona, Mary E. Costanza, Joann L. Wagner

**Affiliations:** ^1^ Meyers Primary Care InstituteWorcesterMAUSA; ^2^ Department of MedicineUniversity of Massachusetts Medical SchoolWorcesterMAUSA; ^3^ University of GeorgiaAthensGAUSA; ^4^ Health Management and PolicyGeorgia State UniversityAtlantaGAUSA; ^5^ Kaiser Permanente GeorgiaAtlantaGAUSA; ^6^ Maine Medical Center Research InstitutePortlandMEUSA; ^7^ Center for Outcomes Research and EvaluationMaine Medical Center Research InstitutePortlandMEUSA; ^8^ Kaiser Permanente Mid‐Atlantic StatesRockvilleMDUSA; ^9^ University of Massachusetts Medical SchoolWorcesterMAUSA

**Keywords:** cancer screening, health literacy, patient engagement, physician–patient communication

## Abstract

**Objective:**

Patient question‐asking is essential to shared decision making. We sought to describe patients' questions when faced with cancer prevention and screening decisions, and to explore differences in question‐asking as a function of health literacy with respect to spoken information (health literacy–listening).

**Methods:**

Four‐hundred and thirty‐three (433) adults listened to simulated physician–patient interactions discussing (i) prophylactic tamoxifen for breast cancer prevention, (ii) PSA testing for prostate cancer and (iii) colorectal cancer screening, and identified questions they would have. Health literacy–listening was assessed using the Cancer Message Literacy Test‐Listening (CMLT‐Listening). Two authors developed a coding scheme, which was applied to all questions. Analyses examined whether participants scoring above or below the median on the CMLT‐Listening asked a similar variety of questions.

**Results:**

Questions were coded into six major function categories: risks/benefits, procedure details, personalizing information, additional information, decision making and credibility. Participants who scored higher on the CMLT‐Listening asked a greater variety of risks/benefits questions; those who scored lower asked a greater variety of questions seeking to personalize information. This difference persisted after adjusting for education.

**Conclusion:**

Patients' health literacy–listening is associated with distinctive patterns of question utilization following cancer screening and prevention counselling. Providers should not only be responsive to the question functions the patient favours, but also seek to ensure that the patient is exposed to the full range of information needed for shared decision making.

## Introduction

Patient engagement, defined as patient involvement in actions needed to obtain the greatest benefit from available health‐care services,^1^ is a key component of patient‐centred care and an important determinant of health status and outcomes.^2^, ^3^, ^4^ Rather than passively receiving health‐care prescriptions and recommendations, engaged patients make affirmative efforts to seek out health information and use that information to make decisions.^5^ Active involvement in discussions with physicians is one mark of patient engagement. This element of engagement with one's provider requires patients to exercise health literacy, which in turn is linked to positive health outcomes.^6^, ^7^

There is considerable overlap between patient engagement activities and the health behaviour patterns ascribed to patients who have high levels of health literacy.^8^ A landmark Institute of Medicine report defined health literacy as including the capacity to obtain and understand both printed and spoken health information, and to apply that information to make health‐related decisions.^9^ Recent conceptualizations of health literacy continue to recognize the central role of oral processing,^10^, ^11^, ^12^ and reviews of research linking health literacy to appropriate utilization of health services and positive health outcomes have decried the fact that most health literacy research has relied on assessments of print literacy only.^13^, ^14^

We have proposed that the capacity to ask questions of physicians (and other health information sources) is a function of ‘interactive health literacy’, that is the kind of health literacy engaged patients enact when they talk with their providers.^15^ Patient question‐asking in clinical encounters is crucial, and is linked to greater comprehension of treatment options^16^ and to greater information provision by physicians.^17^ Limited evidence also suggests an association between patient question‐asking and outcomes such as chronic disease self‐management.^18^

Campaigns have been developed enjoining patients to become more active questioners,^19^ and considerable effort has been devoted to interventions to increase patient question‐asking, but with mixed results so far.^20^, ^21^, ^22^, ^23^ Patients facing decisions involving unfamiliar procedures may need to ask questions to clarify those aspects of the decision which matter most to them, as physicians may not provide complete information.^24^ Patient question‐asking is also critical when the complexity of relevant information about the benefits, harms and uncertainties associated with the available options makes decision making even more challenging.^25^ Such complexity may cause both physicians^26^ and patients^27^ to forego providing and seeking relevant information and to diminish their engagement in shared decision making.

Relatively little is known about patients' capacity for effective question‐asking in cancer screening and prevention,^28^ or about the relationship between patient question‐asking in clinical encounters and patients' ability to understand spoken health information. The ability to understand spoken health information is a key component of health literacy, which we refer to as health literacy–listening. The recent development of an instrument to measure comprehension of spoken information about cancer prevention and screening, the Cancer Message Literacy Test‐Listening (CMLT‐Listening),^29^, ^30^ has enabled new research in this area.

The purpose of this study was to examine question‐asking during decision making about cancer screening and prevention. Of particular interest was the relationship between question‐asking and health literacy–listening. While there is some evidence that patients with lower health literacy reading skills tend to ask fewer questions,^31^, ^32^ we were unable to identify any studies examining whether question‐asking varies for patients at different levels of health literacy‐listening skills, an aspect of health literacy expected to be more closely related to question‐asking. Specifically, we sought to describe the types of questions that analogue patients asked following three simulated discussions, and to explore the relationship between health literacy and the variety of questions generated.

## Methods

### Study population and setting

This study was conducted in the context of the HMO Cancer Research Network (CRN), which consists of the research programmes, enrollee populations and databases of 14 member organizations comprising the HMO Research Network. The CRN's overall goal is to conduct collaborative research to determine the effectiveness of preventive, curative and supportive interventions for major cancers among diverse populations and health systems. The CRN is funded by the National Cancer Institute (U19 CA 079689).

This study was nested within a parent study focused on the development and psychometric evaluation of a test to assess health literacy with respect to spoken information (health literacy‐listening). For the parent study, recruitment targeted a stratified random sample of adult health plan members who had been enrolled for at least 5 years and were aged 40–70. This age range was chosen because of the increased likelihood that patients in this age range would face decisions about cancer prevention and screening, and the fact that cancer risk increases with age. Sampling strata were defined on geocoded United States Census‐based estimates of educational level; at one site, in Atlanta, Georgia, sampling was further stratified to ensure that African American and white members were invited in equal numbers within each educational strata. A variety of recruitment methods were used including mailings, telephone follow‐up and offering study sessions at multiple locations. The invitations described the study as focusing on communication, including physician–patient communication. A cash incentive was provided. Interested members were screened to confirm the ability to communicate in English, adequate corrected hearing and vision, and the absence of any physical or psychological limitations that would interfere with participation. A total of 1074 participants completed interviews in the first round of data collection from 22 June 2009 to 19 April 2010.

For the second round of data collection, 789 participants from the parent study were invited to return to complete a second study session. Invitees were drawn from three health plans: Kaiser Permanente Georgia (KPGA) Atlanta, GA, Kaiser Permanente Hawaii (KPHI) Honolulu, HI, and Fallon Community Health Plan (FCHP) Worcester, MA. One site participating in the parent study did not participate in the second round of data collection due to funding constraints. As in the first round of data collection, recruitment efforts included mail and telephone invitations, reference to physician–patient communication and a cash incentive. Interviews for the second round of data collection occurred between 04 August 2011 and 27 January 2012 and lasted approximately 1–1.5 h. All participants provided written informed consent prior to participation in each study session and were able to withdraw at any time.

### Data collection

All study sessions were conducted in‐person by trained research staff, with oversight from the principal investigator at the study site. Interviewers were provided detailed written instructions, and the online data collection programme (REDCap)^33^ included the interview text for reference during the interview.

### Assessment of health literacy–listening

During the first round of data collection, participants completed the Cancer Message Literacy Test‐Listening (CMLT‐ Listening) which ass‐esses comprehension of spoken health messages related to cancer prevention and screening. This test has strong psychometric properties (e.g. coefficient alpha* *= 0.83) and validity evidence.^29^, ^30^ Health literacy is most often conceived as a stable trait of patients and consumers,^34^ contingent in large measure on socio‐economic status, and unlikely to change over time without deliberate intervention or by modifying the health‐care system.^35^

### Physician–patient vignettes to stimulate question‐asking

Data in this study were elicited by three cancer‐related audio‐vignettes, developed to stimulate question‐asking. The vignettes portrayed physician–patient discussions about three clinical situations and were created by the study team, which included three physicians. The situations were as follows: (i) consideration of tamoxifen for primary prevention of breast cancer in women at elevated risk, (ii) discussion of prostate‐specific antigen (PSA) testing, and (iii) recommendation for colorectal cancer (CRC) screening, with a description of faecal occult blood test (FOBT) and colonoscopy as screening options. Due to the nature of the decision being discussed, the information provided by the physician differed across the vignettes; vignette duration ranged from 2 min, 27 sec to 5 min 11 sec. Both men and women listened and responded to all three clinical situations. Multiple versions of each vignette were created to portray subtle variations in certain physician communication patterns (e.g. whether the physician repeated the main points). Those variations resulted in no differences in relevant outcomes, and these versions were therefore ignored for this analysis. Study materials (vignette texts and print materials) are available upon request from the first author.

Vignette order was randomized for each participant. After listening to each vignette, participants were asked the following: ‘Imagine the doctor is sitting here with us. What questions would you have for him?’ Participants, functioning as analogue patients as is common in patient–provider research,^36^, ^37^ could offer as many questions as they liked, but were prompted for up to three questions. Responses were transcribed verbatim.

### Content analysis of participants' questions

Two authors (DLR and KMM) reviewed a sample of participants' questions and developed preliminary content coding categories. They then reviewed additional questions and independently applied the preliminary codes. Discrepancies were discussed, coding categories were refined, and additional questions were reviewed and coded in an iterative fashion until they agreed that the coding categories were sufficiently defined and captured the relevant elements of participants' questions.

Questions were coded into major categories reflecting six question functions: (i) assessing risks and benefits, (ii) asking for details of procedures, (iii) asking how the information applied to one's personal situation, (iv) asking for additional information beyond the information the doctor introduced in the vignette discourse, (v) asking about the locus of decision making and (vi) establishing the credibility of the physician or other sources (See Table 1). These six functions were arrived at by a combination of *a priori* reasoning and inductive methods. For example, the *a priori* rationale for the *personalizing* function is that a key component of health literacy is not just to acquire information, but to be able to act on information to make personal health decisions.^38^ In contrast, the function *asking for details about the procedures* was established inductively from the numerous instances of participants asking about what they would experience if they underwent the procedure or began preventive treatment. This coding scheme emerged quite similar to one used in an earlier study of CRC screening conversations.^24^

**Table 1:** Question‐function categories and descriptions

Question‐function category	Brief description and exemplary subfunctions
Risks and benefits	Asking about costs and benefits of treatment or screening; includes questions about test accuracy, dangers of procedures, side‐effects of treatment, survival rates and prognosis
Details of the procedures	Asking for additional information, greater detail or clarification of what treatment or screening procedure is actually like; what one would have to do to comply
Applying information to one's personal situation	Asking how the information applies to oneself, or bringing in one's own prior knowledge and asking how that applies
Information external to the doctor's discourse	Asking for new or additional information that goes beyond what was introduced in the vignette; for instance, questions about alternatives
Locus and timing of decision making	Asking what the doctor recommends, for additional information (e.g. books) to inform the decision, or about postponing the decision
Source credibility	Asking about the physician's expertise or experience, asking about credibility of the science or the role of pharma

The six question‐function categories are each comprised of several subcategories reflecting discrete constituent question types or subfunctions. Thus, for example, in the tamoxifen vignette, questions that personalize information can fall into six possible question types or subfunctions (e.g. personal risk of negative side‐effects: ‘What are my personal risks of experiencing negative side‐effects from tamoxifen?’ Or, personal degree of protection against breast cancer: ‘In my individual case, what is the likelihood that tamoxifen would be able to reduce my chances of getting breast cancer?’). Likewise, in the CRC screening vignette, six question types comprise the risk/benefit function (e.g. accuracy/sensitivity of the various screening techniques: ‘Does the other test [FOBT] find polyps like colonoscopy can?’ Or, discomforts of CRC screening: ‘Does that laxative have side‐effects?’). Due to differences in the content of the three vignettes (e.g. the CRC vignette posed the choice between two different screening tests, the PSA vignette focused on just one), the total number of constituent question subfunctions varied. The coding scheme for tamoxifen contained 22 question subfunctions distributed across the six functions; PSA had 24 question subfunctions, and CRC screening 29. The complete coding scheme is available from the first author.

When the coding scheme was finalized, a research assistant (RA) was trained in the coding system. The RA and one author (DR) independently coded a sample of at least 10% of the responses within each of the three vignettes to check coding consistency. Discrepancies were discussed, and double coding of responses continued until coding consistency (exact match in assigning question types for each response) exceeded 80%. The RA then coded the remaining responses. Because many participants' responses were difficult to parse into syntactically delineated questions, we did not compute the number of questions asked. Rather, each response was coded for the presence or absence of each of the constituent question subtypes. Repetitions of question subfunctions within a response to a vignette were not recorded. All participant utterances in response to the probe, ‘What questions would you have?’, were considered in this coding system, irrespective of their grammatical form. We adopted this approach to avoid confounding our outcome (question‐function variety) with the particular linguistic forms in which participants framed their information seeking. For similar reasons, the dependent variables reflect whether a patient used a particular question function rather than how frequently she or he used it.

### Statistical analyses

First, to summarize the variety of question functions used, we computed a variable reflecting whether the participant asked *any* questions under each of the six functions across the three vignettes. For instance, if a participant asked one or more questions in the *personalization* function category in the tamoxifen vignette, one or more questions in the *risk/benefit* function category in the PSA and CRC vignette, and no other questions under any of the other four functions, the participant would be assigned a score of two on this variable.

Second, using the number of subfunction codes within each function used across the three vignettes, we computed the percentage used of the total number available as a measure of *question variety within function*. For example, if a participant asked a question about the risk of breast cancer, another about how one might reduce the likelihood of tamoxifen side‐effects, no questions related to risks or benefits of PSA testing, and a question about the benefits of polyp removal during colonoscopy, the number of risk/benefits subfunction codes applied across the three vignettes would be four of a possible 14 risk/benefit subfunction codes available, resulting in a percentage of 28.6. We also computed the *total number of subfunction* codes utilized across all functions and vignettes (theoretic range, 0–75).

We used the median CMLT‐Listening score to split the sample into two groups, referred to here as high CMLT‐Listening and low CMLT‐Listening. Thus, in this study the designations ‘high’ and ‘low’ are relative terms rather than a criterion‐based classification based on a pre‐determined cut score. We used *t*‐tests to examine the relationship between health literacy and variety in question‐asking. If we detected a significant effect, we also used linear regression models, adjusting for level of education, to assess whether the relationship between question variety and CMLT‐Listening was still present after taking education into account. Education was considered the most important potential moderator to investigate, as measured health literacy often covaries with years of schooling.^39^ Finally, we used a chi‐squared statistic to examine whether participants scoring high or low on the CMLT‐Listening differed in whether they had asked *any* questions. To examine whether the findings depended on the specific cut score used, we repeated all analyses comparing those scoring in the bottom quartile on the CMLT‐Listening to those scoring in the top three quartiles.

Because two of the vignettes contained gender‐specific clinical content, we also examined the effects of gender. We first tested for gender‐related differences in CMLT‐Listening scores using *t*‐tests. We then compared question variety overall, and within each question function (e.g. risk/benefit), again using *t*‐tests. We also computed the percentage of women and men asking questions within each function and subfunction by vignette type.

This study was reviewed and approved by the Institutional Review Board at each site.

## Results

A total of 433 adults participated in the present study and provided usable responses for these analyses. Participant characteristics are presented in Table 2. The mean CMLT‐Listening score for this sample was 79.9, and the standard deviation (SD) was 14.1; scores ranged from 33.3 to 100, with a median of 84.4. We found no statistically significant gender‐related differences in mean CMLT‐Listening scores (*P* > 0.05).

**Table 2:** Participant characteristics

Characteristic	*n*	Percent
Total sample	433	100.0
Gender		
Female	245	56.6
Male	188	43.4
Age in years		
40–49	73	16.9
50–59	158	36.5
60+	202	46.7
Race/ethnicity		
Black/African American	63	14.7
Asian/Native Hawaiian/Pacific Islander	47	10.9
White/Caucasian	277	64.6
Hispanic	15	3.5
American Indian/Alaskan Native	4	0.9
Multiple races	20	4.6
Not reported	3	0.7
Education		
≤ High school or trade school	102	23.7
Some college–graduate school	328	76.3
CMLT‐Listening Score (Mean, SD)	433	79.9 (14.1)
Self‐rated health		
Good/fair/poor	194	44.9
Excellent/very good	238	55.1

Examples of participants' questions, grouped by function and vignette, are presented in Table 3. Table 3 includes descriptive data showing the frequency of utilization of each question function for the three cancer vignettes and overall, the entire sample, and separately for women and men. For example, a substantial majority of participants overall (70%) asked at least one risk/benefit question; the corresponding percentages for women and men were 68% and 73%, respectively.

**Table 3:** Question‐function usage and illustrative quotes by vignette

Function	Risk/Benefits 70% of participants asked at least one risk/benefit question (Women: 68%; Men: 73%)
Clinical Situation Examples	Tamoxifen – *46*% of participants asked at least one risk/benefit question following the tamoxifen vignette. (Women: 42%; Men: 51%) *I probably would like to know more specifics about the percentages of side effects; he gave the percentage of risk reduction via the tamoxifen, 50% reduction in cancer, what are the potential increases in side effect? [Above Median]* …that's scary, whether you take it or not, take the pill, right, or not, it's not a guarantee, the side effects are worse than cancer. [Below Median]
Clinical Situation Examples	PSA – 25% of participants asked at least one risk/benefit question following the PSA vignette (Women: 25%; Men: 25%). …if I do have a PSA test and I either get a positive or a false positive, do you recommend a biopsy? Is there any downside in getting a biopsy? [Above Median] I would ask him…the prostate surgery. I would ask him about the sexual side effects, that would be something, I guess that would stop a lot of guys from having it and that would probably be it…. [Below Median]
Clinical Situation Examples	CRC – 42% of participants asked at least one risk/benefit question following the CRC vignette. (Women: 43%; Men: 40%) When he said rarely, but some side effects, well if you happened to do the colonoscopy and you do get the tearing or the bleeding what can be done or what do they do about that? Then he said that there are pluses and minuses for both, what are they? Well, I got the minuses for the colonoscopy with the tearing and the bleeding. But what are the pluses and minuses for the stool samples? [Above Median] What are the chances of the colonoscopy tearing my lining? Um, and how accurate is that FIT, the stool test, how accurate is it? Because I'm weighing my odds… [Below Median]

Function	Clarification and Details 53% of participants asked at least one clarification and details question (Women: 53%; Men: 53%)
Clinical Situation Examples	Tamoxifen – 19% of participants asked at least one clarification and details question following the tamoxifen vignette. (Women: 17%; Men: 21%) When would I have to start the medication? What would happen if I wanted to continue further than the 5 years which he said was the duration of the prescription? How would I be screened for the side effects? [Above Median] …Is there any time where can you take it for 1 year and then stop or if there is a reaction to these pills like they said what do you do after that?… [Below Median]
Clinical Situation Examples	PSA – 19% of participants asked at least one clarification and details question following the PSA vignette. (Women: 19%; Men: 20%) How long does it take to get results from the blood test? If it were positive it might be painful and I would like to have that explained exactly what do they do that's so painful? [Above Median] …when you get the PSA test results, is there a number that indicates a high PSA number or a low PSA number, or is it just… how do you tell how high your score is? Like is the higher the PSA number is, is it the more likely you have prostate cancer? How frequently do you take the PSA test? Like every year or if you decide not to have the biopsy, like if your PSA result is high like if you want to just retake it, does your number fluctuate between the time you retake and 6 months to a year from now? [Below Median]
Clinical Situation Examples	CRC – 34% of participants asked at least one clarification and details question following the CRC vignette. (Women: 35%; Men:32%) What kind of sedative…what kind of anesthesia whatever…would I be given for the procedure? How long would the procedure last? What happens if polyps are found? [Above Median] Well I guess if it's the first time, I guess I would wanna know how long it's gonna affect me; as far as if there's a discomfort or hurt. And if you find something when you do it, are you gonna go ahead and try to get the samples then? Or later? How bad is the stuff gonna taste you're gonna give me to take? [Below Median]

Function	Applying Information to One's Personal Situation 56% of participants asked at least one question that applied information to their personal situation (Women: 55%; Men: 57%)
Clinical Situation Examples	Tamoxifen – 28% of participants asked at least one question that applied information to their personal situation following the tamoxifen vignette. (Women: 33%; Men:23%) Where could I get more information about the drug and about breast cancer….and more information as I age? She's 48 and she said for 5 years but he said as she gets older their odds go up getting it. So what are my odds at 60? … [Above Median] what is my risk of catching, of getting breast cancer? How often should I be checked if I'm at high risk? Are there things I can do to prevent, you know, my risk of getting breast cancer? Do you, if someone in your family has breast cancer does that mean that I will fall into that same category? [Below Median]
Clinical Situation Examples	PSA – 27% of participants asked at least one question that applied information to their personal situation following the PSA vignette. (Women:22%; Men: 34%) What's the likelihood of me having cancer based on my specific age? … [Above Median] …what would his feelings about getting the test – at my condition, my age and you being my doctor, what decision is best? [Below Median]
Clinical Situation Examples	CRC – 23% of participants asked at least one question that applied information to their personal situation following the CRC vignette. (Women: 22%; Men: 25%) I guess I would want a little more understanding about the chances of getting colon cancer. Just given what he knows about my history and lifestyle or whatever. [Above Median] …why do I have to get tested for that, and am I at risk? …if it runs in my family, and if not why do I have to take the test, or should I take it? Is it common in women, mostly, or is it men, like how common is colon cancer for women? [Below Median]

Function	Information Beyond that Discussed 46% of participants asked at least one question about information beyond that discussed (Women: 44%; Men: 48%)
Clinical Situation Examples	Tamoxifen – 28% of participants asked at least one question about information beyond that discussed following the tamoxifen vignette. (Women: 27%; Men: 29%) How long has the drug been in use? Are there research studies that have been gathered from clinical trials and what not? Where can I read more about tamoxifen? Are there other options? How long would I probably have to take it? And so what are the statistics involving the side effects? I've heard that you could have your breasts removed, and is that a viable option for me at my age and with my level of risk? [Above Median] If there is anything I could do outside of taking medication…like changing my diet or are there things that I should avoid to keep from getting breast cancer other than medication. [Below Median]
Clinical Situation Examples	PSA – 20% of participants asked at least one question about information beyond that discussed following the PSA vignette. (Women: 20%; Men:21%) I probably want to find out if there are any other tests on the horizon, you know, with the genetic stuff that's happening now right now, whether they are developing any additional tests for prostate cancer, and if so I'd want to wait until those tests came out since prostate cancer grows so slowly. [Above Median] … Well I would ask him if is there anything we can do to continue it to be a slow growth such as dietary restraints or such as some type of exercise or some type of physical exercise in order to keep it at a slow growth rate or staying away from alcohol or something like that… [Below Median]
Clinical Situation Examples	CRC – 16% of participants asked at least one question about information beyond that discussed following the CRC vignette (Women:15%; Men: 17%) He just mentioned the colonoscopy or whatever and the blood tests. But, I thought there were other options, like that that sigmoidosocpy, flexible sigmoidoscopy. I would ask him if there were other procedures… [Above Median] I'd ask him is that a certain age people breakdown at 50 years old? [Below Median]

Function	Locus of Decision Making 52% of participants asked at least one question about decision making (Women: 54%; Men: 50%)
Clinical Situation Examples	Tamoxifen – 20% of participants asked at least one question about decision making following the tamoxifen vignette. (Women: 21%; Men: 18%) … I would ask him what would he recommend after giving me all this information? I would say I would want to think about what you told me. When I'm impacted with a lot of information, I don't like to make snap decisions. I would ask him if it's ok if I can think about it for a while, if it's ok that I don't tell you right now what I want to do. [Above Median] What does he recommend? [Below Median]
Clinical Situation Examples	PSA – 35% of participants asked at least one question about decision making following the PSA vignette. (Women: 35%; Men: 35%) I would ask him if he really thinks I should have the test, and if I do have a positive reading, what should I go from there, what should I do? … [Above Median] … I would ask if there was literature available… I would get myself educated before I made another appointment and made any further decisions. I'd write down all my questions after I did my reading. [Below Median]
Clinical Situation Examples	CRC – 18% of participants asked at least one question about decision making following the CRC vignette. (Women: 19%; Men: 16%) …I guess I might wonder what was the evidence‐based recommendation for screening; method of screening? What has the best…as opposed to asking what he would recommend, I'd say what is the general recommendation because that's based on studies. [Above Median] Which would he recommend; the colonoscopy or the fecal sample? [Below Median]

Function	Credibility 35% of participants asked at least one question about credibility (Women: 37%; Men: 32%)
Clinical Situation Examples	Tamoxifen – 12% of participants asked at least one question about credibility following the tamoxifen vignette. (Women: 11%; Men: 13%) That pill doesn't deserve to be around. Good for the drug company and that's it. [Above Median] What is your success rate in preventing breast cancer using tamoxifen? [Below Median]
Clinical Situation Examples	PSA – 14% of participants asked at least one question about credibility following the PSA vignette. (Women: 15%; Men: 12%) … It seemed like he doesn't know what he's saying. He doesn't know what to say. He's saying all kind of things that are contradictory. So he has to chose…It doesn't give me confidence in him…I could probably google it and get the same information he just told me. …I don't trust him already. [Above Median] I would be almost tempted to ask him to decide which side of his mouth he was talking out of because it was like listening to a monologue by two different people; one who was encouraging you to get the test and the other one who is at the same time discouraging you from getting it. [Below Median]
Clinical Situation Examples	CRC – 17% of participants asked at least one question about credibility following the CRC vignette. (Women:18%; Men: 15%) He was pretty clear about the procedure so I don't know if I would…And I'd probably ask if there's someone who's done the procedure a lot, try to get somebody that's skilled at it. [Above Median] Why wasn't he forthcoming on preparation and the patient was asking the preparation? He ignored that altogether, until she asked. [Below Median]

We found no difference in question variety as defined by the total number of different question functions used by health literacy level. The median was three for both groups (*P* > 0.05); the mean for participants scoring below the median on the CMLT‐Listening test was 3.06 function types (SD = 1.3), and the mean for those scoring above the median was 3.17 (SD = 1.5). The total number of question subfunctions utilized also did not differ significantly between the two groups; a mean of 5.2 subfunctions (SD = 2.9) were utilized by those scoring below the median compared to 5.6 (SD = 3.4) by those scoring above the median (*P* > 0.05). Comparisons using the lower cut score (i.e. the bottom quartile) also found no statistically significant differences between the two groups on these measures (*P* > 0.05*)*. Comparisons of these two measures for women and men identified no statistically significant differences (*P* > 0.05).

Table 4 presents results reflecting the variety of question subtypes used within each question function. Participants scoring above the median on the CMLT‐Listening tended to ask a greater variety of questions related to risks and benefits across the three vignettes than participants scoring below the median. This effect was significant after adjusting for education using a linear regression model. When both education and the dichotomous variable based on the CMLT‐Listening median split were entered into the equation, only the coefficient for CMLT‐Listening variable was statistically significant (*P* = 0.038); the *P*‐value associated with the education variable did not approach statistical significance (*P* = 0.37). The effect size associated with the CMLT‐Listening (Cohen's *f* ^2^) was 0.020 and would be characterized as small. However, using the lower cut score, the difference between groups on variety of risk/benefit questions was not statistically significant (*P* > 0.05). In contrast, participants scoring below the median on the CMLT‐Listening tended to ask a greater variety of questions related to personalizing the information. Again, this effect persisted after adjusting for education in linear regression models. When both education and the dichotomous variable based on the CMLT‐Listening median split were entered into the equation, only the coefficient associated with the CMLT‐Listening variable was statistically significant (*P* = 0.002); the *P*‐value associated with the education variable was not significant (*P* = 0.83). The effect size associated with the CMLT‐Listening was small (Cohen's *f* ^2^ = 0.0267). Defining the two groups using the lower cut score also resulted in a statistically significant between‐group difference (*P* = 0.002), and that difference remained statistically significant after adjusting for education. When both education and the dichotomous variable based on the CMLT‐Listening lower cut score were entered into the equation, only the coefficient CMLT‐Listening variable was statistically significant (*P* = 0.001); the *P*‐value associated with education variable was not (*P* = 0.90). Again, the effect size associated with the CMLT‐Listening was small (Cohen's *f* ^2^ = 0.0288).

**Table 4:** Variety in question‐asking: average percentage of subfunctions used

Question function	Participants with CMLT‐Listening scores Below the median (*N* = 215)	Participants with CMLT‐Listening scores Above the median (*N* = 218)	*t*‐test *P*‐value
Risks and benefits	9.8%	12.6%	0.005
Details of the medical procedures	8.8%	10.2%	–
Applying information to one's personal situation	7.3%	4.9%	0.001
Information external to the doctor's discourse	5.1%	6.0%	–
Locus and timing of decision making	5.3%	5.6%	–
Source credibility	5.1%	6.0%	–

No differences were found between participants with high and low CMLT‐Listening scores with respect to question variety on the other four question functions; this was also true when the lower cut score was used. All *P*‐values for these comparisons were > 0.05.

No statistically significant differences between men and women were detected on the variety of question subtypes used within each question function; all *P*‐values associated with these independent *t*‐tests were > 05.

## Discussion

The findings from this study suggest that most patients, regardless of listening health literacy level, are willing and able to generate questions about cancer prevention and screening when prompted to do so. Further, the overall variety of questions generated – that is, the number of different functions that participants' questions addressed – did not vary across the two groups. These findings suggest that low and high listening health literacy patients share many of the same uncertainties when faced with decisions about cancer prevention and screening, and both harbour similar categories of questions.

While we found considerable similarities across high and low listening health literacy participants, we did find two important and statistically significant differences. First, we found that higher literacy participants (as operationalized by the CMLT‐Listening test), relative to their lower literacy counterparts, tended to ask a greater variety of questions focused on the risk and benefits of the procedure or medication under consideration. This finding is consistent with studies linking low health literacy with poor appreciation of health risk analyses,^40^ and suggests that it may be helpful for providers to prompt lower literacy patients to seek out and consider general risk/benefit information about cancer screening or prevention, as they are less likely to spontaneously request it.^41^ On the other hand, higher listening health literacy patients may desire rich information about risk/benefit analyses; messages well adapted to this group therefore would offer more extensive risk information. Because identifying patients who are at lower literacy levels may be challenging for clinicians,^42^ it may be helpful for clinicians to be trained in methods for eliciting questions from all patients, as has been suggested previously.^43^, ^44^

Second, we found that lower listening health literacy participants tended to ask a greater variety of questions seeking to personalize the information, compared to higher literacy participants. This finding persisted across two different cut scores, as well as after adjusting for education using linear regression. Rather than seeking general information about cancer screening and prevention, lower listening health literacy patients desired information that was directly tailored to their particular circumstances, including their personal and family health histories. Thus, while higher literacy participants asked questions like, ‘How often does a faecal exam miss finding a real case of cancer?’, lower literacy participants were more likely to ask, ‘If my stool sample is clear of cancer, do I still have to do the colonoscopy?’ This intriguing finding has several potential explanations that call for further research. One possibility is that low literacy individuals have greater difficulty understanding probability estimates or the concept of chance, or that low‐ and high‐literacy individuals rely on different interpretations of probability. For example, in a study of patients with cancer considering participation in phase I trials, Weinfurt *et al*.^45^ found that patients with less education endorsed belief‐type interpretations of probability estimates ‘The doctor is 40% confident that the treatment will control my cancer’, while patients with greater education endorsed frequency‐type interpretations (e.g. ‘For every 100 patients like me, the treatment will work for 40 patients'). More research is needed to test these and alternative explanations of our findings, and to determine ways to help lower literacy patients understand how information about a decision applies to their individual circumstances.

The six major question‐function categories, applicable across all three vignettes, provide new insights into what considerations and topics are most salient to patients when they think about cancer prevention and screening. Overall, questions related to understanding the risks and benefits of the procedure were most broadly utilized. As shown in Table 3, 70% of participants asked at least one question about risks/benefits. Not surprisingly, for example, a substantial number of participants asked about tamoxifen side‐effects, or questioned whether the risk reduction obtained with tamoxifen would outweigh the risks of serious side‐effects, consistent with findings reported previously regarding patients' decision making about tamoxifen prophylaxis.^46^, ^47^, ^48^, ^49^ Risk‐/benefit‐related questions for PSA testing tended to focus on the prevalence of prostate cancer or false‐positive or false‐negative errors associated with PSA testing. Risk/benefit questions for CRC screening tended to focus on the advantages and disadvantages of the different screening methods, and their associated risks.

Also noteworthy in the descriptive data conveyed in Table 3 is the finding that questioning about the risks and benefits of PSA screening, the intervention with the greatest associated scientific uncertainty, was relatively infrequent, compared to tamoxifen use or CRC screening. The percentage of participants asking risk/benefit questions after each vignette was 25%, 46% and 42% for PSA, tamoxifen and CRC screening, respectively. On the other hand, questions about the locus of decision making were most highly utilized following the PSA discussion. The percentage of participants asking questions about the locus of decision making following each vignette was 35%, 20% and 18% for PSA, tamoxifen and CRC screening, respectively.

Many participants responded to the PSA vignette by seeking an opinion or direct recommendation from the physician, and several expressed dissatisfaction that the physician in the vignette did not offer more directive advice. This pattern of attempting to defer to the physician's judgment is consistent with the high levels of uncertainty surrounding PSA discussions.

Not surprisingly, participants were more likely to ask procedure‐related questions after the CRC screening conversation, compared to the tamoxifen and PSA screening conversations. Colonoscopy procedure requires patients to prepare themselves ahead of time, and many questions focused on that preparation and subsequent return to routine. Usage of questions related to personalizing the information was relatively similar across the three cancer vignettes; in all three scenarios, participants sought information on how the information would apply to them, with their particular characteristics or history. As mentioned above, however, seeking out personalized information of this nature was most pronounced among participants who exhibited low health literacy.

It is noteworthy that the measure of health literacy that distinguished question generating preferences between people with high and low levels of health literacy was based on oral communication. Measurement of health literacy has become a major pre‐occupation, but most measures remain reading‐based.^50^ The CMLT‐Listening is one of a small and relatively recent set of instruments that acknowledges the primacy of clinical information transmission through oral interaction, and so would be expected to be more strongly related to information exchange in clinical encounters than conventional reading‐based instruments.

This study had limitations. First, participants were reacting to a simulated rather than an actual conversation in which they were participating. While there are advantages to this approach (i.e. the presentation was standardized), there are also disadvantages in that participants had no opportunity to actually interact with the physician and were not given an opportunity to ask questions until the end of the conversation. Further, the psycho‐social influences which may serve to inhibit or facilitate question‐asking differed from those in actual clinical encounters. It is possible that participants felt more able to ask questions in this study, as some inhibiting factors, such as time pressure on the physician, were not present, and that such inhibiting effects might interact with health literacy in real encounters. In addition, the use of simulation precluded exploration of whether physician–patient concordance in terms of gender, race, ethnicity or socio‐economic position affected question‐asking, an important but unanswered question. Our use of this simulated situation may have resulted in more question‐asking than would have occurred in an actual encounter, as we explicitly encouraged question‐asking. Use of analogue patients in research on clinical communication is quite common and, while different from actual patients in live encounters, can yield valuable insights.^36^, ^37^ However, we were not able to explore additional research investigating the role of the factors in this study (such as whether lower literacy patients are more inhibited in real encounters), and it is clearly needed. We also note that the CMLT‐Listening, our measure of health literacy, was administered at an earlier study session. At present, there is no information available on the stability of CMLT‐Listening scores over time. As in any study where volunteers are recruited, it is not known how representative these participants' responses are with respect to the general population. For example, many study participants had a college education, suggesting that this sample was more educated than the population overall. A final, but important, limitation is the absence of a measure of prior knowledge about the three interventions discussed in the vignettes.

One of the key issues that has emerged in the field of health messaging pertains to the relative merits of tailoring information to relevant patient individual differences,^51^ as opposed to utilizing a, ‘universal precautions’^52^ approach in health messages. Thus, for example, some studies offer limited support for providing detailed statistics about cancer screening and prevention to persons exhibiting pronounced ‘need for cognition’.^53^ In contrast, the trend in health literacy practices leans towards creating messages that can be easily processed by individuals with limited health literacy, in part because of the difficulty of accurately screening patients' health literacy levels in clinical settings.^54^ Some have argued, however, that certain health literacy practices, when applied universally, can actually deprive consumers and patients of risk information they might wish to know.^55^ Clearly, more research is needed to determine optimum practices for increasing patient question‐asking in decision making and to examine the effects of training providers in best practices.

## Conclusion

Patient question‐asking is a critical component of both patient engagement and health literacy, and a prerequisite to shared decision making. This study provides insight into the types of questions patients may have when presented with information about three cancer prevention and screening decisions. The greater variety of personalizing questions asked by patients with lower health literacy‐listening scores is intriguing and warrants replication and further exploration.

## Funding

This study was funded by a grant from the National Cancer Institute titled Cancer Research Network (CRN) Across Health Care Systems (U19 CA079689). Dr. Mazor currently receives funding from a subsequent grant, the Cancer Research Resources & Collaboration in Integrated Health Care Systems U24 CA171524, which funds the fourth cycle of the CRN. The CRN welcomes collaboration with external investigators and institutions. For more information about how to collaborate with the CRN, please visit crn.cancer.gov. The funder had no role in study design, data collection, analysis and interpretation of data, writing of this manuscript or the decision to submit this paper for publication. Dr. Cutrona is supported by the National Center for Advancing Translational Sciences of the National Institutes of Health under award number KL2TR000160. The content is solely the responsibility of the authors and does not necessarily represent the official views of the NIH.

## Conflict of interest

None of the authors has any conflicts of interest to report.
